# Mobility-related brain regions linking carotid intima-media thickness to specific gait performances in old age

**DOI:** 10.1186/s12877-024-04918-1

**Published:** 2024-04-01

**Authors:** Xin Zhang, Heyang Lu, Min Fan, Weizhong Tian, Mei Cui, Yanfeng Jiang, Chen Suo, Tiejun Zhang, Kelin Xu, Yingzhe Wang, Xingdong Chen

**Affiliations:** 1 School of Public Health, the Key Laboratory of Public Health Safety of Ministry of Education, Fudan University, Shanghai, China; 2 Fudan University Taizhou Institute of Health Sciences, Taizhou, Jiangsu China; 3 Department of Neurology, Huashan Hospital, Fudan University, Shanghai, China; 4 Taixing Disease Control and Prevention Center, Taizhou, Jiangsu China; 5 Taizhou People’s Hospital Affiliated to Nantong University, Taizhou, Jiangsu China; 6 State Key Laboratory of Genetic Engineering, Zhangjiang Fudan International Innovation Center, School of Life Sciences, Human Phenome Institute, Fudan University, Shanghai, China; 7 National Clinical Research Center for Aging and Medicine, Huashan Hospital, Fudan University, Shanghai, China; 8 Yiwu Research Institute of Fudan University, Yiwu, Zhejiang China

**Keywords:** Brain structure, Carotid intima-media thickness, Gait, Mediation analysis

## Abstract

**Background:**

Gait disturbance is common in older adults with vascular diseases. However, how carotid atherosclerosis affects gait remains poorly understood. The objectives were to investigate the associations between carotid intima-media thickness and specific gait performances and explore the potential role of brain structure in mediating these associations.

**Methods:**

A cross-sectional analysis of data from the Taizhou Imaging Study was conducted, including 707 individuals who underwent both gait and carotid ultrasound examinations. Gait assessments include the Timed-Up-and-Go test, the Tinetti test, and quantitative gait assessment using a wearable device. Quantitative parameters were summarized into independent gait domains with factor analysis. Magnetic resonance images were obtained on a 3.0-Tesla scanner, and the volumes of fifteen brain regions related to motor function (primary motor, sensorimotor), visuospatial attention (inferior posterior parietal lobules, superior posterior parietal lobules), executive control function (dorsolateral prefrontal cortex, anterior cingulate), memory (hippocampus, entorhinal cortex), motor imagery (precuneus, parahippocampus, posterior cingulated cortex), and balance (basal ganglia: pallidum, putamen, caudate, thalamus) were computed using FreeSurfer and the Desikan-Killiany atlas. Mediation analysis was conducted with carotid intima-media thickness as the predictor and mobility-related brain regions as mediators.

**Results:**

Carotid intima-media thickness was found to be associated with the Timed-Up-and-Go performance (β = 0.129, *p* = 0.010) as well as gait performances related to pace (β=-0.213, *p* < 0.001) and symmetry (β = 0.096, *p* = 0.045). Besides, gait performances were correlated with mobility-related brain regions responsible for motor, visuospatial attention, executive control, memory, and balance (all FDR < 0.05). Notably, significant regions differed depending on the gait outcomes measured. The primary motor (41.9%), sensorimotor (29.3%), visuospatial attention (inferior posterior parietal lobules, superior posterior parietal lobules) (13.8%), entorhinal cortex (36.4%), and motor imagery (precuneus, parahippocampus, posterior cingulated cortex) (27.3%) mediated the association between increased carotid intima-media thickness and poorer Timed-Up-and-Go performance. For the pace domain, the primary motor (37.5%), sensorimotor (25.8%), visuospatial attention (12.3%), entorhinal cortex (20.7%), motor imagery (24.9%), and balance (basal ganglia: pallidum, putamen, caudate, thalamus) (11.6%) acted as mediators.

**Conclusions:**

Carotid intima-media thickness is associated with gait performances, and mobility-related brain volume mediates these associations. Moreover, the distribution of brain regions regulating mobility varies in the different gait domains. Our study adds value in exploring the underlying mechanisms of gait disturbance in the aging population.

**Supplementary Information:**

The online version contains supplementary material available at 10.1186/s12877-024-04918-1.

## Introduction

Gait disturbance is a prevalent chronic disorder among community-dwelling older adults, leading to an increased risk of falls, morbidity, and mortality [1–3]. Previous studies have shown that an increased cardiovascular risk is associated with impaired gait function, specifically decreased gait speed [4, 5]. Carotid atherosclerosis, primarily measured by carotid intima-media thickness (IMT), is associated with a higher risk of gait disorders, such as decreased walking speed and impaired balance ability [6–8]. This association between cardiovascular health and gait may be attributed to neurological abnormalities. According to the heart-brain axis, cardiovascular diseases (CVDs) could impair cerebral microvascular function [9]. For example, functional degeneration and structural changes in the carotid arteries profoundly affect intracranial blood vessels, resulting in brain structural changes such as white matter lesions [10] and brain atrophy [11, 12]. These changes in white and gray matter [13, 14], particularly brain volume [15–17], have been associated with gait disorders. Additionally, volume loss in brain regions responsible for mobility exhibits varying associations with poorer gait performances [18, 19], which might be attributed to low perfusion in different regions responsible for specific aspects of gait.

Nevertheless, few studies focus on the mechanisms through which carotid atherosclerosis impacts gait, and it remains unclear whether brain structure plays a vital role. Notably, previous studies that investigated the association between carotid atherosclerosis and gait have commonly used individual indicators or clinical rating scales to measure gait. However, in terms of specificity and sensitivity, sensor-based measurement techniques outperformed traditional methods. They can help obtain vast amounts of quantitative gait data in different dimensions and unveil gait problems in different domains [20, 21]. In this study, we used similar techniques to capture specific gait patterns.

Therefore, further understanding of how carotid atherosclerosis affects gait could help develop interventions to improve gait performances in the aging population. With objective quantitative gait analysis, this population-based study examined the associations of carotid IMT and plaque with specific gait performances. We also investigated whether mobility-related brain regions could mediate this relationship. We hypothesize that certain mobility-related brain regions may mediate the association between carotid indicators and specific gait performances.

## Methods

### Study design and participants

We conducted a cross-sectional study based on the Taizhou Imaging Study (TIS), an ongoing community-based cohort study. The inclusion and exclusion criteria for the baseline population have been described in detail previously [22]. Among the 904 individuals included in TIS, 741 participants underwent gait analysis. After excluding individuals with incomplete gait data, current use of mobility aids, incomplete carotid ultrasound examination, and incomplete magnetic resonance imaging (MRI) data, 707 individuals were included in the current analysis (Fig. S1). Ethical approval for the TIS was obtained from the Ethics Committee of the School of Life Sciences, Fudan University, and Fudan University Taizhou Institute of Health Sciences (Institutional Review Board approval numbers: 496 and B017, respectively). Written informed consent was obtained from all participants prior to data and biospecimen collection.

### Measurement of gait

Gait assessment involved both clinical rating scales and quantitative gait assessment. The clinical rating scales assessment consisted of two commonly used tests: the Timed-Up-and-Go (TUG) test [23] and the Tinetti test [24]. The TUG test evaluates basic mobility and balance abilities by measuring the time it takes for an individual to complete a set of subtasks, such as standing up from a chair, walking 3 m, and turning around. On the other hand, the Tinetti test is a questionnaire that comprises 17 items in two subscales, with nine items assessing balance and eight items evaluating gait. Each item is scored 0–1 or 0–2, where a score of 0 represents the poorest performance. The total score of the Tinetti test ranges from 0 to 28. These standardized tests are widely utilized to assess the mobility and functional capacities of individuals in a clinical setting.

The quantitative gait data was collected through an insole-style wearable gait tracking device (Senno gait, Sennotech Co. Ltd., China). Participants were instructed to start from a designated point and walk a distance of 10 m in a straight line. They were then required to return along the same path to the starting point. During the whole process, participants were asked to walk at a comfortable and self-selected pace. The gait tracking device recorded and analyzed various gait parameters for each stride, excluding the turning strides. The quantitative gait parameters measured included stride time, stance time, swing time, stance time percentage of the gait cycle (%GC) symmetry, swing time (%GC) symmetry, stride time symmetry, stride time coefficient of variation (CV), stance time CV, swing time CV, heel strike angle, stride length, maximum swing velocity, gait velocity, stance time (%GC), and double support time (%GC). Considering that these spatiotemporal gait parameters are highly correlated, we subsequently summarized them into independent gait domains by factor analysis. We provide the definitions of each gait parameter and the domains they reflected a priori and indicate the direction in which a parameter is considered “poorer” (Table S1). According to previous studies, the impaired TUG test was defined as the duration of the TUG test > 12 s [25], and the impaired gait velocity was defined as gait velocity < 1 m/s [26]. The impaired gait group was defined by gait velocity < 1.0 m/s or TUG test duration > 12 s.

### Measurement of carotid IMT and plaque

Carotid ultrasound examinations were conducted using the Philips iE33 Ultrasound System. Participants were positioned in a supine position to ensure the neck area was fully exposed for measurement. The optimal segment for measurement was identified as 10 mm proximal and distal to the carotid bulb, with the measurement taken on the distal walls. Bilateral common and internal carotid IMT measurements were recorded for each subject, and the maximum value from the four sites was used for analysis [27]. Carotid plaque was defined based on one of the three conditions: a focal protrusion in the vascular lumen exceeding 0.5 mm, a thickness 50% greater than the surrounding IMT, or an IMT exceeding 1.5 mm [28]. The ultrasound examinations were performed by the same technician using the same instrument, and computer software was used to collect the relevant features, ensuring completion of the examination within three cardiac cycles.

### MRI acquisition and measurement

All participants underwent MRI scans using a 3.0T scanner (Magnetom Verio Tim scanner; Siemens, Erlangen, Germany) at Taizhou People’s Hospital. The detailed protocol and sequence parameters for MRI have been previously reported [29]. This study used volumetric measures of specific brain regions associated with mobility as regions of interest for analysis. Fifteen brain regions were selected a priori, including motor function (primary motor, sensorimotor), visuospatial attention (inferior posterior parietal lobules, superior posterior parietal lobules), executive control function (dorsolateral prefrontal cortex, anterior cingulate), memory (hippocampus, entorhinal cortex), motor imagery (precuneus, parahippocampus, posterior cingulated cortex), and balance (basal ganglia: pallidum, putamen, caudate, thalamus), based on previous studies [18, 19]. FreeSurfer software (v6.0.0) was used to extract the volume data of functional brain regions from structural T1 images, utilizing the Desikan-Killiany atlas [30]. All brain region volume was standardized using z-scores for analysis.

### Co-variables

Demographic and lifestyle characteristics data were collected using a comprehensive questionnaire, which included information on age, sex, smoking habits, alcohol consumption, and physical activity. Physical examinations and diagnoses conducted by physicians obtained additional data on height and weight, and the body mass index (BMI) information was calculated as mass in kilograms divided by height in meters squared. Blood pressure was measured in person by experienced technicians at the Taizhou People’s Hospital, and morning fasting blood samples were collected by a certified nurse and processed using standard protocols [22]. Medical history, including hypertension, diabetes, and hyperlipidemia, were defined as the following criteria. Hypertension was defined as a blood pressure greater than 140/90 mmHg, a self-reported history, or current use of antihypertensive drugs. Diabetes was defined as fasting blood glucose greater than 7.0 mmol/L, a self-reported history, or current use of anti-diabetic drugs. Hyperlipidemia was defined as total cholesterol greater than 5.2 mmol/L, triglyceride greater than 1.7 mmol/L, a self-reported history, or current use of antihyperlipidemic drugs. Current smoking was defined by the frequency of smoking in the past six months and categorized into “never” and ≥ one cigarette every 1–3 days. Current alcohol drinking was defined by the frequency in the past six months and categorized into “never” and ≥ three times per week. Physical activity was defined by the frequency of exercise during the past year, including “never”, “1–3 times per month”, “1–2 times per week”, “3–5 times per week”, and “6–7 times per month”. We categorized physical activity into “no exercise” and “exercise” by classifying “1–3 times per month”, “1–2 times per week”, “3–5 times per week”, and “6–7 times per month” into the “exercise” group and incorporated it as a binary variable in our models. The analysis considered these covariates to account for their potential influence on the relationships among carotid IMT, brain regions, and gait performances.

### Statistical analysis

#### Description analysis

Continuous variables were reported as mean (standard deviation, SD), and categorical variables were presented as frequencies (%). The normality of continuous variables was assessed using the Shapiro-Wilk test. Since the data presented skewness, logarithmic transformation was applied to the TUG, Tinetti, and gait variables related to symmetry, and square root transformation was applied to the gait variables related to variability. Group differences were examined using the following statistical tests: the two-sample t-test or Wilcoxon rank sum test was used for continuous variables, and Pearson’s chi-squared test or Fisher’s exact test was applied for categorical variables.

### Factor analysis of quantitative gait parameters

A factor analysis (varimax rotation, eigenvalue 1.0) was employed for the 15 quantitative gait parameters [30]. The original variables in the rhythm, symmetry, variability, and phase domains were inverted to represent poorer gait performances with lower values. Variables with correlation loadings greater than 0.5 were considered significantly correlated with the corresponding factors. Gait parameters were then categorized into independent gait domains based on the extracted factors, which were standardized and expressed as z-scores for further analysis.

### Association analysis

General linear models were employed to investigate the associations among carotid indicators, gait performances (clinical rating scale performances and gait domains), and mobility-related brain regions. The regression analysis was fitted in two models: Model 1 was adjusted for age, sex, and standardized total intracranial volume (included in the analysis of brain volume); Model 2 was further adjusted for BMI, hypertension, diabetes, hyperlipidemia, smoking, drinking, and physical activity. The dependent and independent variables were standardized before regression analysis, by subtracting the mean and dividing by the SD for each value of each variable. The results of regression were shown as standardized beta coefficients. Multiple testing problem was corrected by the false discovery rate (FDR) method.

### Mediation analysis

Mediation analysis was performed to investigate whether differences in brain region volume mediated the associations between carotid IMT and gait performances. The brain regions that showed statistically significant associations with both carotid IMT and gait performances were included in the analysis. The mediation analysis used the R package “mediation” with 1,000 repetitions to estimate the direct effect (path c’), the mediation effect, and the total effect (path c). Using the R package “medsens”, we performed the sensitivity analysis of mediation to explore whether the presence of unmeasured confounders would violate the assumption and to identify the robustness of the results [31]. The results were presented as ρ, a Pearson correlation between the error terms of the regression models for the mediator and outcome and the product of the coefficient of determination of the two models. The threshold of statistical significance was set at corrected *p* < 0.05. Statistical analyses were performed using R software (Version 4.2.2).

## Results

### Characteristics of the study population

Demographic, cardiovascular risk factors, neuroimaging, and representative gait characteristics of the 707 involved participants are presented in Table 1. The mean age of the participants was 59.8 years, with females accounting for 57.7%. All included subjects were divided into two groups based on gait characteristics. The impaired gait group, comprising 27.7% of the sample (*N* = 196), tended to be older and female. They also exhibited higher BMI, smaller gray matter volume, and a higher prevalence of hypertension.

**Table 1:** Characteristics of study participants

Characteristics	Study population (*N* = 707)	Unimpaired gait (*N* = 511)	Impaired gait (*N* = 196)	*p*
**Demographics**				
Age, years	59.8 (3.1)	59.6 (3.1)	60.2 (3.0)	**0.021**
Female	408 (57.7)	280 (54.8)	128 (65.3)	**0.014**
Height, cm	158.9 (7.8)	159.7 (7.7)	156.9 (7.8)	**< 0.001**
BMI, kg/m^2^	24.44 (3.16)	24.19 (2.92)	25.08 (3.64)	**0.008**
**Cardiovascular risk factors**				
Current smoker	86 (12.2)	69 (13.5)	17 (8.7)	0.101
Alcohol consumption	199 (28.1)	156 (30.5)	43 (21.9)	**0.028**
Physical activity				0.655
Never	599 (84.7)	428 (83.8)	171 (87.2)	
1–3 times/month	8 (1.1)	5 (1.0)	3 (1.5)	
1–2 times/week	23 (3.3)	17 (3.3)	6 (3.1)	
3–5 times/week	28 (4.0)	22 (4.3)	6 (3.1)	
6–7 times/month	49 (6.9)	39 (7.6)	10 (5.1)	
Hypertension	359 (53.8)	244 (51.0)	115 (60.8)	**0.028**
Diabetes mellitus	72 (10.7)	51 (10.5)	21 (11.2)	0.901
Hyperlipidemia	381 (56.3)	277 (56.6)	104 (55.3)	0.822
**Neuroimaging**				
Total intracranial volume, cm^3^	1465.82 (159.62)	1471.23 (158.13)	1451.73 (163.03)	0.146
White matter volume, ml	503.71 (128.08)	503.56 (129.93)	504.09 (123.42)	0.961
Gray matter volume, ml	558.33 (60.59)	562.07 (58.57)	548.57 (64.71)	**0.008**
**Carotid ultrasonography**				
Carotid IMT, mm	0.84 (0.24)	0.84 (0.25)	0.85 (0.20)	0.399
Carotid plaque	178 (25.2)	130 (25.4)	48 (24.5)	0.870

*Note* Categorical variables are presented as numbers (percentages), and continuous variables as means (SDs).

*Abbreviation* BMI, body mass index; IMT, Intima-media thickness; TUG, Timed-Up-and-Go; %GC, percentage of the gait cycle

### Association analysis of carotid IMT and plaque with gait performances

As shown in Table 2, per 1-standard deviation (SD) increase in the carotid IMT was significantly associated with poorer TUG performance (β = 0.153, *p* < 0.001). This association remained robust after adjusting for cardiovascular risk factors (β = 0.129, *p* = 0.010).

**Table 2:** Associations of IMT and plaque with clinical rating scale performances

	Model	TUG test^*^ β (*p*)	Adjusted R^2^	Tinetti test^*^ β (*p*)	Adjusted R^2^
IMT	Model 1	**0.153 (< 0.001)**	0.036	-0.026 (0.531)	-0.001
	Model 2	**0.129 (0.010)**	0.045	-0.007 (0.891)	0.018
Plaque	Model 1	0.101 (0.244)	0.020	0.018 (0.835)	-0.002
	Model 2	0.074 (0.418)	0.030	0.019 (0.841)	0.014

*Note* Standardized regression coefficients (β) and *p* values from linear regression models are presented. Differences significant at *p* < 0.05 are highlighted in bold. Model 1 was adjusted for sex and age; Model 2 was further adjusted for BMI, hypertension, diabetes, hyperlipidemia, smoking, alcohol consumption, and physical activity

*For skewed variables the logarithm is presented

*Abbreviation* IMT, Intima-media thickness; TUG, Timed-Up-and-Go

We summarized the quantitative gait parameters into five independent domains based on factor analysis. The five summarized factors accounted for 89.51% of the total variance (Table S2). These factors were categorized as follows based on previous research [20, 32]: rhythm domain (stride time, stance time, swing time), phase domain (double support time percentage of the gait cycle, stance time percentage of the gait cycle), symmetry domain (stance time percentage of the gait cycle symmetry, swing time percentage of the gait cycle symmetry, stride time symmetry), variability domain (stance time coefficient of variation, swing time coefficient of variation, stride time coefficient of variation), and pace domain (stride length, maximum swing velocity, heel strike angle, gait velocity).

The associations of carotid IMT and plaque with gait domains were further examined (Table 3). After full adjustment, carotid IMT was found to be associated with specific gait domains, including symmetry (β = 0.096, *p* = 0.045) and pace (β = -0.213, *p* < 0.001). However, no statistically significant association was observed between carotid plaque and any gait characteristics.

**Table 3:** Associations of gait domains with IMT and plaque

	Model	Rhythm			Symmetry			Phase			Variability			Pace		
		β (95% CI)	*p*	R^2^_adj_	β (95% CI)	*p*	R^2^_adj_	β (95% CI)	*p*	R^2^_adj_	β (95% CI)	*p*	R^2^_adj_	β (95% CI)	*p*	R^2^_adj_
**IMT**	Model 1	-0.007 (-0.087,0.073)	0.864	0.085	**0.077 (0.001,0.153)**	**0.047**	0.002	0.039 (-0.043,0.121)	0.352	0.025	**-0.084 (-0.166, -0.001)**	**0.048**	0.017	**-0.221 (-0.300, -0.143)**	**< 0.001**	0.092
	Model 2	0.006 (-0.082,0.095)	0.887	0.109	**0.096 (0.002,0.190)**	**0.045**	0.001	0.033 (-0.064,0.130)	0.501	0.050	-0.086 (-0.180, 0.008)	0.074	0.025	**-0.213 (-0.304, -0.121)**	**< 0.001**	0.117
**Plaque**	Model 1	0.003 (-0.162,0.167)	0.973	0.079	0.003 (-0.169,0.175)	0.971	-0.002	0.006 (-0.164,0.175)	0.947	0.023	0.020 (-0.151, 0.191)	0.819	0.009	0.082 (-0.086, 0.249)	0.339	0.048
	Model 2	0.010 (-0.153,0.173)	0.903	0.095	0.001 (-0.188,0.189)	0.996	-0.001	0.043 (-0.136,0.222)	0.639	0.048	0.074 (-0.099, 0.248)	0.399	0.019	0.114 (-0.060, 0.287)	0.198	0.080

*Note* Standardized regression coefficients (β) and *p* values from linear regression models are presented. Differences significant at *p* < 0.05 are highlighted in bold. Model 1 was adjusted for sex and age; Model 2 was further adjusted for BMI, hypertension, diabetes, hyperlipidemia, smoking, alcohol consumption, and physical activity

*Abbreviation* IMT, Intima-media thickness; CI, confidence interval

### Association analysis of carotid IMT and gait performances with mobility-related brain regions

**Fig. 1:**
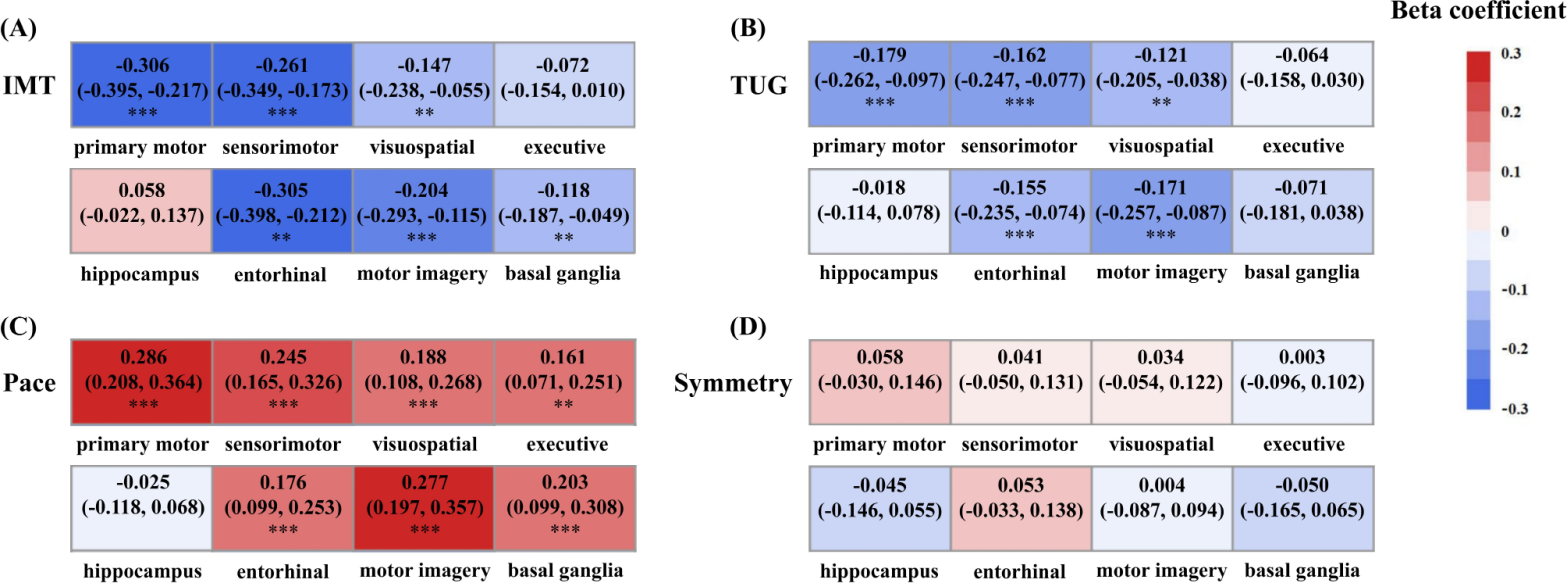
Associations of IMT and IMT-related gait performances with mobility-related brain regions. (Standardized regression coefficients (β) and significance from linear regression models are presented. All models were adjusted for sex, age, standardized total intracranial volume, BMI, hypertension, diabetes, hyperlipidemia, smoking, alcohol consumption, and physical activity. The significance threshold was set at *p < 0.05, **p < 0.01, and ***p < 0.001 after correcting for the false discovery rate.)

Figure 1A and Table S3 illustrate that the significant regions associated with carotid IMT encompassed most mobility-related regions. However, the executive control function and hippocampus did not show significant associations (β = -0.072 and 0.058, respectively; both FDR > 0.05).

After full adjustment, poorer TUG performance was significantly associated with smaller volume in the primary motor (β = -0.179, FDR < 0.001), sensorimotor (β = -0.162, FDR < 0.001), visuospatial attention (β = -0.121, FDR = 0.007), entorhinal cortex (β = -0.155, FDR < 0.001), and motor imagery (β = -0.171, FDR < 0.001) regions (Fig. 1B and Table S4). Additionally, the pace domain exhibited positive correlations with the volume of the primary motor (β = 0.286, FDR < 0.001), sensorimotor (β = 0.245, FDR < 0.001), visuospatial attention (β = 0.188, FDR < 0.001), executive control (β = 0.161, FDR = 0.001), entorhinal cortex (β = 0.176, FDR < 0.001), motor imagery (β = 0.277, FDR < 0.001), and basal ganglia (β = 0.203, FDR < 0.001) regions (Fig. 1C and Table S4). However, no statistically significant association was observed between mobility-related brain volume and the symmetry domain (Fig. 1D and Table S4). Except for the two domains related to increased IMT, we also found the variability domain was associated with the volume of the primary motor (β = 0.129, FDR = 0.007), sensorimotor (β = 0.148, FDR = 0.004), and entorhinal cortex (β = 0.103, FDR = 0.026) (Table S5).

### The mediation effect of mobility-related brain regions in the associations between carotid IMT and gait performances

**Fig. 2:**
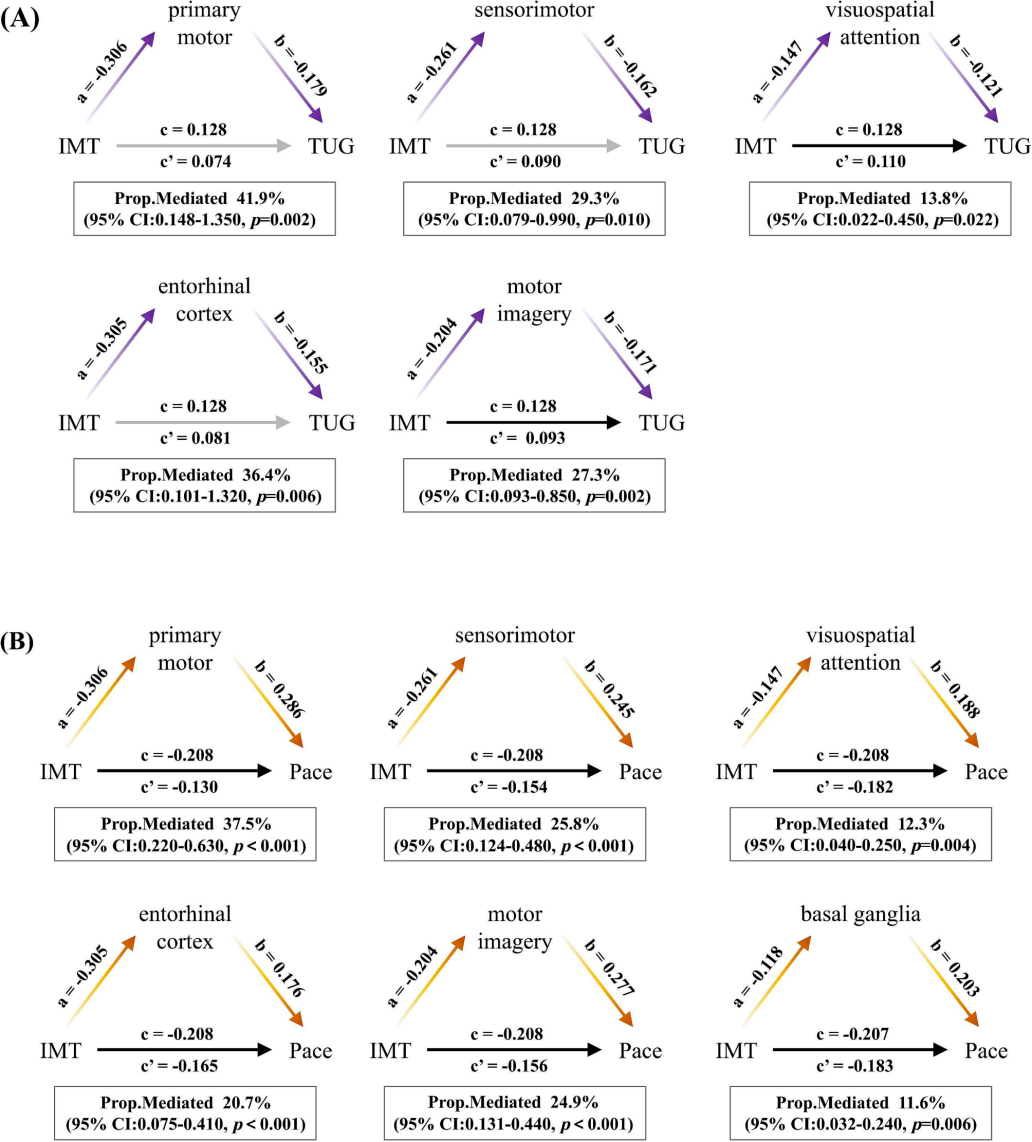
Mediation effects of mobility-related brain regions in the associations between IMT and specific gait performances. (All models were adjusted for sex, age, BMI, hypertension, diabetes, hyperlipidemia, smoking, alcohol consumption, and physical activity. Standardized total intracranial volume was added when brain volume was included. CI, confidence interval.)

We investigated whether mobility-related brain regions mediate the relationships between carotid IMT and gait performances using mediation analysis. As presented in Fig. 2A and Table S6, we found areas associated with primary motor (41.9%, *p*_mediation_=0.002), sensorimotor (29.3%, *p*_mediation_=0.010), visuospatial attention (13.8%, *p*_mediation_=0.022), entorhinal cortex (36.4%, *p*_mediation_=0.006), and motor imagery (27.3%, *p*_mediation_=0.002) mediated the relationship between carotid IMT and TUG performance. Additionally, the primary motor (37.5%, *p*_mediation_<0.001), sensorimotor (25.8%, *p*_mediation_<0.001), visuospatial attention (12.3%, *p*_mediation_=0.004), entorhinal cortex (20.7%, *p*_mediation_<0.001), motor imagery (24.9%, *p*_mediation_<0.001), and basal ganglia (11.6%, *p*_mediation_=0.006) mediated the association between carotid IMT and the pace domain (Fig. 2B and Table S6).

### Sensitivity analysis

We conducted sensitivity analyses to assess the potential impact of unmeasured confounding in the mediation analysis. The results, displayed in Table S7, indicated that the estimated indirect effects were relatively robust to the presence of unmeasured confounders, as indicated by the sensitivity parameter (ρ). Considering the effect of different exercise frequencies, we repeated the analysis by using physical activity as an ordinal variable instead of a binary one, where no significantly different results were found (Table S8–S14).

## Discussion

This study investigated potential mechanisms underlying the relationships among increased carotid intima-media thickness, mobility-related brain volume, and gait abnormalities in community-dwelling Chinese older adults. Our major findings are as follows. First, increased carotid IMT was independently associated with poorer gait performances, whereas carotid plaque did not show any significant associations with gait disturbance. Second, poorer gait performances were linked to smaller volumes of mobility-related brain regions, and the significant regions differed depending on the gait outcomes measured. Finally, the volume of mobility-related brain regions mediated the relationship between increased carotid IMT and gait disturbance.

In alignment with our findings, previous studies have consistently shown that carotid IMT affects gait performance [6–8]. Notably, we found that carotid IMT was positively correlated with the symmetry domain. It may be attributed to the biological mechanism of cerebrovascular disease, which can lead to diffuse lesions affecting bilateral neural pathways and then increase gait symmetry [33]. The association between carotid plaque and poor gait performance has been reported [6, 34]. However, our study did not find significant associations between carotid plaque and other gait domains, such as rhythm and phase. These discrepancies might be due to differences in the study population and gait measurements. Our study involved relatively younger and healthier individuals compared to the previous research. In addition to the TUG test and Tinetti test, our research used more objective quantitative gait measures, whereas the other studies we compared used the 40-foot walking test, maximum walking speed, and the modified Tinetti scale alone.

The significant regions differed depending on the gait parameter measured, which may be due to impairment of the underlying circuits controlling pace, variability, and balance. For instance, it has been shown that slower gait speed is associated with selective volume of the motor cortex, parahippocampus, and dorsolateral prefrontal cortex, which play a vital role in motor, motor imagery, and executive control functions [18, 35]. Furthermore, higher step length variability was associated with the volume of the hippocampus, superior parietal lobe, and anterior cingulate gyrus, which are crucial for maintaining gait stability and consistency [36]. Additionally, the volume of the superior lobule and putamen, involved in visuospatial orientation and regulation, may lead to poorer balance control [35]. Interestingly, we found that brain regions affecting the variability domain differed from those affecting the pace domain, indicating that mobility-related brain regions control different gait domains [37]. The pace domain was associated with almost all mobility-related areas, suggesting that the excessively sensitive pace regulation might involve widespread gray matter networks. The variability domain was associated with the primary motor, sensorimotor cortex, and entorhinal cortex. The involvement of the entorhinal cortex is noteworthy as it is an early affected region in Alzheimer’s disease pathology [38], implying the importance of cognitive factors in influencing gait variability. However, our results focused on two specific gait domains, and further research is needed to explore whether the brain-gait association is indeed specific to these domains or extends to other domains of gait.

Our current investigation demonstrated that brain regions related to motor, visuospatial attention, memory, motor imagery, and balance mediate the relationships between carotid IMT and gait performances. Regretfully, research investigating the effects of brain structure on the association between atherosclerosis and gait remains limited. Nevertheless, some indirect studies shed light on the role of neural factors in this relationship. For example, gray matter atrophy, common in individuals with carotid atherosclerosis [12], has been reported to have detrimental effects on specific gait changes [14, 39]. The potential disturbance of local cerebral microcirculation of gray matter can help explain the findings in the current study. Of its high metabolic demand and great cerebral blood flow, the gray matter may be impacted by increased carotid IMT. Damage to essential fiber bundles and loops in these regions can impair gait-related functions.

Our study provides novel insights into the mediating effects of mobility-related brain regions on the relationships between carotid IMT and specific gait performances in old age. We employed a quantitative approach to measure gait function, which offers greater sensitivity than qualitative or semi-quantitative methods. We could conduct a focused analysis of specific gait patterns by categorizing highly correlated gait parameters into different domains. Moreover, we explored the combined effects of vascular factors and the nervous system on gait and identified potential mechanisms. However, our study has several limitations. First, it is challenging to establish a direct causative sequence as a cross-sectional study. Further investigation using longitudinal designs of TIS would be valuable in unraveling the causal relationships among carotid IMT, mobility-related brain regions, and gait performances. Second, musculoskeletal factors, such as muscle mass and strength, osteopenia, and osteoarthritis, were not taken into account. Including these factors in future studies would provide a more comprehensive understanding of gait. Third, our study only focused on the mediating role of gray matter volume between carotid IMT and gait. Considering the mediating role of white matter health in the relationship between CVDs and gait, future studies need to consider whether white matter integrity mediates the association between carotid atherosclerosis and gait performances. Finally, considering the structural and functional connectivity between brain regions, further analysis is warranted to reveal how the functional dynamics between these regions relate to carotid atherosclerosis and gait performances.

## Conclusion

The present study indicates that increased carotid IMT is associated with the volume of mobility-related brain regions, leading to poor gait performances. And, different gait domains are affected by mobility-related brain regions responsible for specific functions. Our findings highlight the specific contribution of gray matter volume to gait disturbance associated with increased carotid IMT. However, larger population studies and longitudinal investigations are necessary to validate our findings further and elucidate the neural mechanisms underlying the association between carotid atherosclerosis and gait disturbance in more detail.

### Electronic supplementary material

Below is the link to the electronic supplementary material.

Supplementary Material 1

Supplementary Material 2

Supplementary Material 3

Supplementary Material 4

Supplementary Material 5

Supplementary Material 6

Supplementary Material 7

Supplementary Material 8

Supplementary Material 9

Supplementary Material 10

Supplementary Material 11

Supplementary Material 12

Supplementary Material 13

Supplementary Material 14

Supplementary Material 15

## Data Availability

The datasets used and/or analysed during the current study are available from the corresponding author on reasonable request.

## Abbreviations

BMI: Body mass index
CI: Confidence interval
CV: Coefficient of variation
CVDs: Cardiovascular diseases
FDR: False discovery rate
IMT: Intima-media thickness
MRI: Magnetic resonance imaging
SD: Standard deviation
TIS: Taizhou Imaging Study
TUG: Timed-Up-and-Go
%GC: Percentage of the gait cycle
