# Supplementary figures and images for: Mobility-related brain regions linking carotid intima-media thickness to specific gait performances in old age
Source: BMC Geriatr. 2024 Apr 1;24:303. doi: 10.1186/s12877-024-04918-1 (PMC10983675; doi:10.1186/s12877-024-04918-1)

**
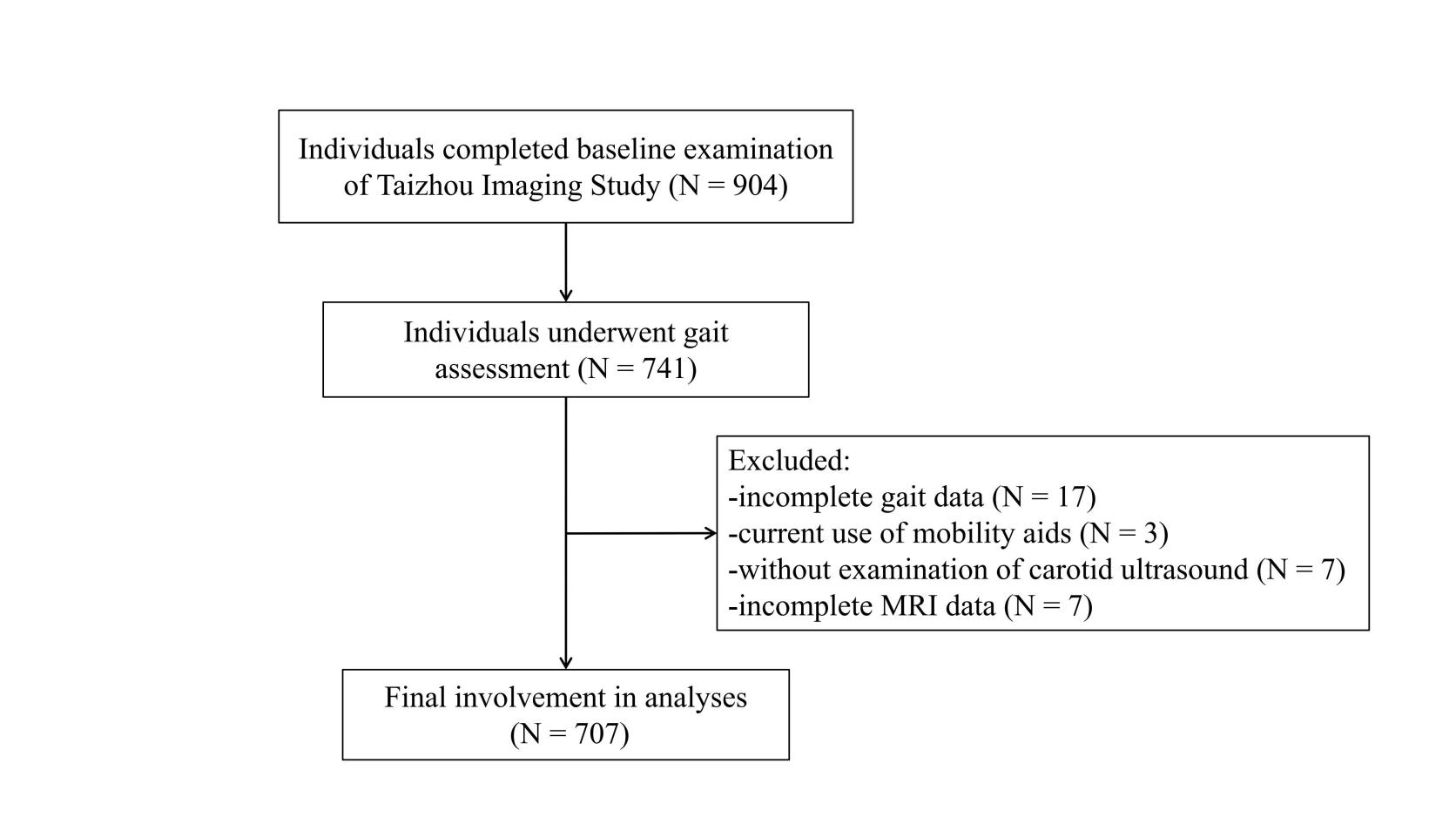
**

**Figure S1. Flowchart of the participants recruitment in this study.**

Supplement: Supplementary file 1 — Supplementary Material 1 [file 12877_2024_4918_MOESM1_ESM.docx]
