# Supplementary material for: Mobility-related brain regions linking carotid intima-media thickness to specific gait performances in old age
Source: BMC Geriatr. 2024 Apr 1;24:303. doi: 10.1186/s12877-024-04918-1 (PMC10983675; doi:10.1186/s12877-024-04918-1)
Supplement: Supplementary file 2 — Supplementary Material 2 [file 12877_2024_4918_MOESM2_ESM.docx]

| **Table S1. Definition of gait parameters.** | | | |
| --- | --- | --- | --- |
| Parameter | Description | Indication of “poorer” gait | Domain |
| Stride time | The time between the first contacts of two consecutive footfalls of the same foot. | Higher | Rhythm |
| Stance time | The time of the cycle during which part of the foot touches the ground. It begins when the foot first touches the ground and ends when the same foot leaves the ground. | Higher | Rhythm |
| Swing time | The time of the cycle during which the foot is in the air and does not touch the ground. It begins when the foot first leaves the ground and ends when the same foot touches the ground again. | Higher | Rhythm |
| Stance time %GC symmetry | The ratio of the higher stance time %GC to the lower. | Higher | Symmetry |
| Swing time %GC symmetry | The ratio of the higher swing time %GC to the lower. | Higher | Symmetry |
| Stride time symmetry | The ratio of the higher stride time to the lower. | Higher | Symmetry |
| Stance time CV | The coefficient of variation of stance time. | Higher | Variability |
| Swing time CV | The coefficient of variation of swing time. | Higher | Variability |
| Stride time CV | The coefficient of variation of stride time. | Higher | Variability |
| Heel strike angle | The angle between the foot and the ground at heel strike, on a vertical plane. | Lower | Pace |
| Stride length | The distance between two consecutive footprints on the ground, parallel to the line of progression. | Lower | Pace |
| Maximum swing velocity | The maximum forward speed of the foot during swing. | Lower | Pace |
| Gait velocity | The mean speed of forward walking, calculated in meters per second. | Lower | Pace |
| Stance time (%GC) | The percentage of the stance time in the gait cycle. | Higher | Phase |
| Double support time (%GC) | The percentage of the double support time in the gait cycle. | Higher | Phase |
| Abbreviation: CV, coefficient of variation; %GC, percentage of the gait cycle. | | | |
