# Supplementary material for: Mobility-related brain regions linking carotid intima-media thickness to specific gait performances in old age
Source: BMC Geriatr. 2024 Apr 1;24:303. doi: 10.1186/s12877-024-04918-1 (PMC10983675; doi:10.1186/s12877-024-04918-1)
Supplement: Supplementary file 3 — Supplementary Material 3 [file 12877_2024_4918_MOESM3_ESM.docx]

| **Table S2. Factor analysis of gait parameters** | | | | | | |
| --- | --- | --- | --- | --- | --- | --- |
| Gait Parameter | | **Rhythm** | **Symmetry** | **Phase** | **Variability** | **Pace** |
| **Rhythm** |  | |  |  |  |  |
| Stride time, ms | **0.924** | | 0.179 | 0.125 | 0.233 | 0.161 |
| Stance time, ms | **0.871** | | 0.159 | -0.446 | 0.038 | 0.067 |
| Swing time, ms | **0.836** | | 0.071 | 0.437 | 0.205 | 0.183 |
| **Symmetry** |  | |  |  |  |  |
| Stance time %GC symmetry | 0.104 | | **0.942** | -0.109 | 0.190 | 0.099 |
| Swing time %GC symmetry | 0.090 | | **0.905** | 0.037 | 0.267 | 0.117 |
| Stride time symmetry | 0.191 | | **0.824** | -0.113 | 0.290 | 0.075 |
| **Phase** |  | |  |  |  |  |
| Double support time (%GC), % | 0.045 | | -0.090 | **0.963** | 0.134 | 0.138 |
| Stance time (%GC), % | 0.058 | | -0.078 | **0.962** | 0.111 | 0.156 |
| **Variability** |  | |  |  |  |  |
| Stance time CV, % | 0.211 | | 0.245 | 0.116 | **0.890** | 0.162 |
| Swing time CV, % | 0.211 | | 0.245 | 0.116 | **0.890** | 0.162 |
| Stride time CV, % | 0.021 | | 0.327 | 0.102 | **0.714** | 0.141 |
| **Pace** |  | |  |  |  |  |
| Stride length, m | 0.078 | | 0.201 | 0.364 | 0.172 | **0.820** |
| Maximum swing velocity, m/s | 0.455 | | 0.136 | 0.182 | 0.067 | **0.802** |
| Heel strike angle, ° | -0.132 | | -0.042 | -0.226 | 0.349 | **0.743** |
| Gait velocity, m/s | 0.437 | | 0.163 | 0.339 | 0.032 | **0.685** |
| **% of variance** | 40.48 | | 19.44 | 12.99 | 9.26 | 7.34 |
| Note: Correlation loadings greater than 0.5 are highlighted in bold.  Abbreviation: CV, coefficient of variation; %GC, percentage of the gait cycle. | | | | | | |
