# Supplementary material for: Mobility-related brain regions linking carotid intima-media thickness to specific gait performances in old age
Source: BMC Geriatr. 2024 Apr 1;24:303. doi: 10.1186/s12877-024-04918-1 (PMC10983675; doi:10.1186/s12877-024-04918-1)
Supplement: Supplementary file 6 — Supplementary Material 6 [file 12877_2024_4918_MOESM6_ESM.docx]

| **Table S5. Associations of mobility-related brain regions with variability domain.** | | | | | |
| --- | --- | --- | --- | --- | --- |
| Mobility-related regions | Model | Variability | | | |
|  |  | β (95%CI) | *p* | *p* (FDR) | R^2^_adj_ |
| Primary motor | Model 1 | 0.149 (0.074, 0.224) | <0.001 | **<0.001** | 0.028 |
|  | Model 2 | 0.129 (0.049, 0.209) | 0.002 | **0.007** | 0.032 |
| Sensorimotor | Model 1 | 0.168 (0.090, 0.246) | <0.001 | **<0.001** | 0.032 |
|  | Model 2 | 0.148 (0.066, 0.230) | <0.001 | **0.004** | 0.036 |
| Visuospatial attention | Model 1 | 0.062 (-0.015, 0.140) | 0.112 | 0.180 | 0.010 |
|  | Model 2 | 0.042 (-0.039, 0.124) | 0.305 | 0.488 | 0.018 |
| Executive control function | Model 1 | 0.009 (-0.078, 0.096) | 0.835 | 0.835 | 0.007 |
|  | Model 2 | -0.001 (-0.092, 0.090) | 0.979 | 0.979 | 0.017 |
| Hippocampus | Model 1 | 0.035 (-0.058, 0.128) | 0.459 | 0.525 | 0.009 |
|  | Model 2 | -0.025 (-0.117, 0.068) | 0.601 | 0.687 | 0.018 |
| Entorhinal cortex | Model 1 | 0.110 (0.035, 0.185) | 0.004 | **0.011** | 0.019 |
|  | Model 2 | 0.103 (0.025, 0.181) | 0.010 | **0.026** | 0.027 |
| Motor imagery | Model 1 | 0.064 (-0.012, 0.140) | 0.098 | 0.180 | 0.011 |
|  | Model 2 | 0.047 (-0.037, 0.130) | 0.271 | 0.488 | 0.018 |
| Basal ganglia | Model 1 | 0.061 (-0.041, 0.163) | 0.239 | 0.319 | 0.010 |
|  | Model 2 | 0.031 (-0.074, 0.137) | 0.562 | 0.687 | 0.018 |
| Note: Standardized regression coefficients (β) and FDR-corrected *p* values from linear regression models are presented. Differences significant at FDR-*p* < 0.05 are highlighted in bold. Model 1 was adjusted for sex, age, and standardized total intracranial volume; Model 2 was further adjusted for BMI, hypertension, diabetes, hyperlipidemia, smoking, alcohol consumption, and physical activity.  Abbreviations: CI, confidence interval; FDR, false discovery rate. | | | | | |
