# Supplementary material for: Mobility-related brain regions linking carotid intima-media thickness to specific gait performances in old age
Source: BMC Geriatr. 2024 Apr 1;24:303. doi: 10.1186/s12877-024-04918-1 (PMC10983675; doi:10.1186/s12877-024-04918-1)
Supplement: Supplementary file 7 — Supplementary Material 7 [file 12877_2024_4918_MOESM7_ESM.docx]

| **Table S6. Mediation effects of mobility-related brain regions in the relation between carotid IMT and specific gait performances.** | | | | | |
| --- | --- | --- | --- | --- | --- |
| Outcome | Mediator | Total effect | ADE | ACDE | Proportion mediated |
| TUG | Primary motor | 0.128 (0.045,0.220)** | 0.074 (-0.021,0.170) | 0.054 (0.020,0.100)*** | 0.419 (0.148,1.350)** |
|  | Sensorimotor | 0.128 (0.045,0.220)** | 0.090 (-0.0002,0.180) | 0.037 (0.011,0.070)** | 0.293 (0.079,0.990)** |
|  | Visuospatial attention | 0.128 (0.045,0.220)** | 0.110 (0.025,0.200)** | 0.018 (0.003,0.040)* | 0.138 (0.022,0.450)* |
|  | Entorhinal cortex | 0.128 (0.044,0.220)** | 0.081 (-0.015,0.180) | 0.047 (0.016,0.090)** | 0.364 (0.101,1.320)** |
|  | Motor imagery | 0.128 (0.045,0.230)** | 0.093 (0.009,0.180)* | 0.035 (0.012,0.070)*** | 0.273 (0.093,0.850)** |
| Pace | Primary motor | -0.208 (-0.296,-0.140)*** | -0.130 (-0.218,-0.060)*** | -0.078 (-0.124,-0.050)*** | 0.375 (0.220,0.630)*** |
|  | Sensorimotor | -0.208 (-0.296,-0.140)*** | -0.154 (-0.240,-0.080)*** | -0.054 (-0.091,-0.030)*** | 0.258 (0.124,0.480)*** |
|  | Visuospatial attention | -0.208 (-0.296,-0.140)*** | -0.182 (-0.265,-0.110)*** | -0.026 (-0.053,-0.010)** | 0.123 (0.040,0.250)** |
|  | Entorhinal cortex | -0.208 (-0.296,-0.140)*** | -0.165 (-0.260,-0.090)*** | -0.043 (-0.077,-0.020)*** | 0.207 (0.075,0.410)*** |
|  | Motor imagery | -0.208 (-0.296,-0.140)*** | -0.156 (-0.238,-0.090)*** | -0.052 (-0.093,-0.030)*** | 0.249 (0.131,0.440)*** |
|  | Basal ganglia | -0.207 (-0.297,-0.140)*** | -0.183 (-0.270,-0.110)*** | -0.024 (-0.046,-0.010)** | 0.116 (0.032,0.240)** |
| All models were adjusted for sex, age, standardized total intracranial volume, BMI, hypertension, diabetes, hyperlipidemia, smoking, alcohol consumption, and physical activity. Values are estimated coefficients (95%CI). The significance threshold was set at **p*<0.05, ***p*<0.01, and ****p*<0.001.  Abbreviations: IMT, Intima-media thickness; TUG, Timed-Up-and-Go; ADE, average direct effect; ACME, average causal mediated effect. | | | | | |
