# Supplementary material for: Mobility-related brain regions linking carotid intima-media thickness to specific gait performances in old age
Source: BMC Geriatr. 2024 Apr 1;24:303. doi: 10.1186/s12877-024-04918-1 (PMC10983675; doi:10.1186/s12877-024-04918-1)
Supplement: Supplementary file 8 — Supplementary Material 8 [file 12877_2024_4918_MOESM8_ESM.docx]

| **Table S7. Sensitivity analysis of the mediation effect of ROI volumes in the association of carotid IMT and specific gait performances.** | |
| --- | --- |
| Sensitivity results | Outcomes |
| **Carotid IMT** |  |
|  | **TUG** |
| For primary motor: ρ at which ACME = 0 | -0.158 |
| For sensorimotor: ρ at which ACME = 0 | -0.129 |
| For visuospatial attention: ρ at which ACME = 0 | -0.112 |
| For entorhinal cortex: ρ at which ACME = 0 | -0.144 |
| For motor imagery: ρ at which ACME = 0 | -0.154 |
|  | **Pace** |
| For primary motor: ρ at which ACME = 0 | 0.242 |
| For sensorimotor: ρ at which ACME = 0 | 0.195 |
| For visuospatial attention: ρ at which ACME = 0 | 0.172 |
| For entorhinal cortex: ρ at which ACME = 0 | 0.141 |
| For motor imagery: ρ at which ACME = 0 | 0.241 |
| For basal ganglia: ρ at which ACME = 0 | 0.152 |
| Abbreviations: IMT, Intima-media thickness; TUG, Timed-Up-and-Go; ACME, average causal mediated effect. | |
