# Supplementary material for: Mobility-related brain regions linking carotid intima-media thickness to specific gait performances in old age
Source: BMC Geriatr. 2024 Apr 1;24:303. doi: 10.1186/s12877-024-04918-1 (PMC10983675; doi:10.1186/s12877-024-04918-1)
Supplement: Supplementary file 9 — Supplementary Material 9 [file 12877_2024_4918_MOESM9_ESM.docx]

| **Table S8.** Associations of IMT and plaque with clinical rating scale performances. | | | | | |
| --- | --- | --- | --- | --- | --- |
|  | Model | TUG test^*^ β (*p*) | Adjusted R^2^ | Tinetti test^*^ β (*p*) | Adjusted R^2^ |
| IMT | Model 1 | **0.153 (<0.001)** | 0.036 | -0.026 (0.531) | -0.001 |
|  | Model 2 | **0.130 (0.009)** | 0.044 | -0.005 (0.929) | 0.020 |
| Plaque | Model 1 | 0.101 (0.244) | 0.020 | 0.018 (0.835) | -0.002 |
|  | Model 2 | 0.081 (0.378) | 0.028 | 0.012 (0.896) | 0.015 |
| Note: Standardized regression coefficients (β) and *p* values from linear regression models are presented. Differences significant at *p* < 0.05 are highlighted in bold. Model 1 was adjusted for sex and age; Model 2 was further adjusted for BMI, hypertension, diabetes, hyperlipidemia, smoking, alcohol consumption, and physical activity (ordinal). *For skewed variables the logarithm is presented.  Abbreviation: IMT, Intima-media thickness; TUG, Timed-Up-and-Go. | | | | | |
