# Supplementary material for: Mobility-related brain regions linking carotid intima-media thickness to specific gait performances in old age
Source: BMC Geriatr. 2024 Apr 1;24:303. doi: 10.1186/s12877-024-04918-1 (PMC10983675; doi:10.1186/s12877-024-04918-1)
Supplement: Supplementary file 10 — Supplementary Material 10 [file 12877_2024_4918_MOESM10_ESM.docx]

| **Table S9.** Associations of gait domains with IMT and plaque. | | | | | | | | | | | | | | | | |
| --- | --- | --- | --- | --- | --- | --- | --- | --- | --- | --- | --- | --- | --- | --- | --- | --- |
|  |  | **Rhythm** | | | **Symmetry** | | | **Phase** | | | **Variability** | | | **Pace** | | |
|  | Model | β (95% CI) | *p* | R^2^_adj_ | β (95% CI) | *p* | R^2^_adj_ | β (95% CI) | *p* | R^2^_adj_ | β (95% CI) | *p* | R^2^_adj_ | β (95% CI) | *p* | R^2^_adj_ |
| **IMT** | Model 1 | -0.007  (-0.087,0.073) | 0.864 | 0.085 | **0.077**  **(0.001,0.153)** | **0.047** | 0.002 | 0.039  (-0.043,0.121) | 0.352 | 0.025 | **-0.084**  **(-0.166, -0.001)** | **0.048** | 0.017 | **-0.221**  **(-0.300, -0.143)** | **<0.001** | 0.092 |
|  | Model 2 | 0.007  (-0.081,0.096) | 0.869 | 0.106 | **0.096**  **(0.002,0.190)** | **0.045** | -0.002 | 0.034  (-0.063,0.132) | 0.488 | 0.047 | -0.082  (-0.176, 0.012) | 0.089 | 0.028 | **-0.213**  **(-0.305, -0.122)** | **<0.001** | 0.123 |
| **Plaque** | Model 1 | 0.003  (-0.162,0.167) | 0.973 | 0.079 | 0.003  (-0.169,0.175) | 0.971 | -0.002 | 0.006  (-0.164,0.175) | 0.947 | 0.023 | 0.020  (-0.151, 0.191) | 0.819 | 0.009 | 0.082  (-0.086, 0.249) | 0.339 | 0.048 |
|  | Model 2 | 0.011  (-0.153,0.175) | 0.894 | 0.092 | -0.003  (-0.191,0.186) | 0.978 | 0.002 | 0.044  (-0.136,0.223) | 0.634 | 0.045 | 0.079  (-0.094, 0.252) | 0.368 | 0.023 | 0.103  (-0.070, 0.276) | 0.243 | 0.084 |
| Note: Standardized regression coefficients (β) and *p* values from linear regression models are presented. Differences significant at *p* < 0.05 are highlighted in bold. Model 1 was adjusted for sex and age; Model 2 was further adjusted for BMI, hypertension, diabetes, hyperlipidemia, smoking, alcohol consumption, and physical activity (ordinal). Abbreviation: IMT, Intima-media thickness; CI, confidence interval. | | | | | | | | | | | | | | | | |
