# Supplementary material for: Mobility-related brain regions linking carotid intima-media thickness to specific gait performances in old age
Source: BMC Geriatr. 2024 Apr 1;24:303. doi: 10.1186/s12877-024-04918-1 (PMC10983675; doi:10.1186/s12877-024-04918-1)
Supplement: Supplementary file 11 — Supplementary Material 11 [file 12877_2024_4918_MOESM11_ESM.docx]

| **Table S10. Associations of mobility-related brain regions with carotid IMT.** | | | | | |
| --- | --- | --- | --- | --- | --- |
| Mobility-related regions | Model | IMT | | | |
|  |  | β (95%CI) | *p* | *p* (FDR) | R^2^_adj_ |
| Primary motor | Model 1 | -0.318 (-0.396, -0.240) | <0.001 | **<0.001** | 0.110 |
|  | Model 2 | -0.306 (-0.395, -0.217) | <0.001 | **<0.001** | 0.188 |
| Sensorimotor | Model 1 | -0.274 (-0.350, -0.197) | <0.001 | **<0.001** | 0.171 |
|  | Model 2 | -0.260 (-0.349, -0.172) | <0.001 | **<0.001** | 0.228 |
| Visuospatial attention | Model 1 | -0.158 (-0.237, -0.078) | <0.001 | **<0.001** | 0.085 |
|  | Model 2 | -0.146 (-0.238, -0.054) | 0.002 | **0.002** | 0.141 |
| Executive control function | Model 1 | -0.089 (-0.160, -0.018) | 0.014 | **0.016** | 0.276 |
|  | Model 2 | -0.074 (-0.156, 0.008) | 0.078 | 0.090 | 0.297 |
| Hippocampus | Model 1 | 0.047 (-0.019, 0.113) | 0.165 | 0.165 | 0.377 |
|  | Model 2 | 0.059 (-0.021, 0.139) | 0.148 | 0.148 | 0.378 |
| Entorhinal cortex | Model 1 | -0.291 (-0.371, -0.210) | <0.001 | **<0.001** | 0.086 |
|  | Model 2 | -0.305 (-0.398, -0.211) | <0.001 | **<0.001** | 0.150 |
| Motor imagery | Model 1 | -0.225 (-0.304, -0.145) | <0.001 | **<0.001** | 0.075 |
|  | Model 2 | -0.204 (-0.293, -0.115) | <0.001 | **<0.001** | 0.142 |
| Basal ganglia | Model 1 | -0.141 (-0.200, -0.083) | <0.001 | **<0.001** | 0.506 |
|  | Model 2 | -0.119 (-0.188, -0.050) | 0.001 | **0.001** | 0.532 |
| Note: Standardized regression coefficients (β) and FDR-corrected *p* values from linear regression models are presented. Differences significant at FDR-*p* < 0.05 are highlighted in bold. Model 1 was adjusted for sex, age, and standardized total intracranial volume; Model 2 was further adjusted for BMI, hypertension, diabetes, hyperlipidemia, smoking, alcohol consumption, and physical activity (ordinal).  Abbreviations: IMT, Intima-media thickness; CI, confidence interval; FDR, false discovery rate. | | | | | |
