# Supplementary material for: Mobility-related brain regions linking carotid intima-media thickness to specific gait performances in old age
Source: BMC Geriatr. 2024 Apr 1;24:303. doi: 10.1186/s12877-024-04918-1 (PMC10983675; doi:10.1186/s12877-024-04918-1)
Supplement: Supplementary file 12 — Supplementary Material 12 [file 12877_2024_4918_MOESM12_ESM.docx]

| **Table S11. Associations of mobility-related brain regions with IMT-related gait performances.** | | | | | | | | | | | | | |
| --- | --- | --- | --- | --- | --- | --- | --- | --- | --- | --- | --- | --- | --- |
| Mobility-related regions | Model | TUG test | | | | Pace | | | | Symmetry | | | |
|  |  | β (95%CI) | *p* | *p* (FDR) | R^2^_adj_ | β (95%CI) | *p* | *p* (FDR) | R^2^_adj_ | β (95%CI) | *p* | *p* (FDR) | R^2^_adj_ |
| Primary motor | Model 1 | -0.176 (-0.250, -0.102) | <0.001 | **<0.001** | 0.046 | 0.310 (0.239, 0.380) | <0.001 | **<0.001** | 0.141 | 0.046 (-0.030, 0.122) | 0.239 | 0.805 | 0.001 |
|  | Model 2 | -0.180 (-0.263, -0.097) | <0.001 | **<0.001** | 0.052 | 0.287 (0.209, 0.364) | <0.001 | **<0.001** | 0.154 | 0.062 (-0.026, 0.150) | 0.165 | 0.528 | 0.007 |
| Sensorimotor | Model 1 | -0.171 (-0.248, -0.093) | <0.001 | **<0.001** | 0.042 | 0.271 (0.196, 0.346) | <0.001 | **<0.001** | 0.114 | 0.031 (-0.049, 0.110) | 0.448 | 0.805 | -0.001 |
|  | Model 2 | -0.161 (-0.246, -0.076) | <0.001 | **<0.001** | 0.045 | 0.242 (0.161, 0.322) | <0.001 | **<0.001** | 0.131 | 0.042 (-0.048, 0.133) | 0.356 | 0.528 | 0.005 |
| Visuospatial attention | Model 1 | -0.116 (-0.192, -0.040) | 0.003 | **0.005** | 0.029 | 0.201 (0.127, 0.275) | <0.001 | **<0.001** | 0.086 | 0.031 (-0.046, 0.109) | 0.431 | 0.805 | -0.001 |
|  | Model 2 | -0.121 (-0.204, -0.037) | 0.005 | **0.008** | 0.036 | 0.186 (0.106, 0.266) | <0.001 | **<0.001** | 0.112 | 0.038 (-0.050, 0.127) | 0.396 | 0.528 | 0.005 |
| Executive control function | Model 1 | -0.073 (-0.159, 0.013) | 0.097 | 0.129 | 0.020 | 0.175 (0.091, 0.259) | <0.001 | **<0.001** | 0.071 | 0.010 (-0.077, 0.097) | 0.825 | 0.943 | -0.002 |
|  | Model 2 | -0.063 (-0.157, 0.031) | 0.188 | 0.228 | 0.026 | 0.161 (0.071, 0.251) | <0.001 | **0.001** | 0.100 | 0.006 (-0.093, 0.105) | 0.909 | 0.909 | 0.004 |
| Hippocampus | Model 1 | -0.023 (-0.116, 0.069) | 0.619 | 0.619 | 0.018 | -0.052 (-0.143, 0.039) | 0.259 | 0.259 | 0.051 | -0.026 (-0.120, 0.067) | 0.575 | 0.805 | -0.001 |
|  | Model 2 | -0.018 (-0.114, 0.078) | 0.715 | 0.715 | 0.026 | -0.026 (-0.119, 0.066) | 0.575 | 0.575 | 0.083 | -0.048 (-0.148, 0.053) | 0.351 | 0.528 | 0.005 |
| Entorhinal cortex | Model 1 | -0.157 (-0.231, -0.084) | <0.001 | **<0.001** | 0.040 | 0.226 (0.154, 0.298) | <0.001 | **<0.001** | 0.098 | 0.050 (-0.026, 0.126) | 0.194 | 0.805 | 0.001 |
|  | Model 2 | -0.158 (-0.238, -0.077) | <0.001 | **<0.001** | 0.046 | 0.181 (0.104, 0.259) | <0.001 | **<0.001** | 0.113 | 0.058 (-0.027, 0.143) | 0.182 | 0.528 | 0.007 |
| Motor imagery | Model 1 | -0.161 (-0.235, -0.086) | <0.001 | **<0.001** | 0.041 | 0.275 (0.203, 0.346) | <0.001 | **<0.001** | 0.121 | 0.001 (-0.075, 0.077) | 0.979 | 0.979 | -0.002 |
|  | Model 2 | -0.171 (-0.256, -0.085) | <0.001 | **<0.001** | 0.047 | 0.274 (0.194, 0.354) | <0.001 | **<0.001** | 0.144 | 0.006 (-0.085, 0.096) | 0.901 | 0.909 | 0.004 |
| Basal ganglia | Model 1 | -0.073 (-0.174, 0.029) | 0.160 | 0.183 | 0.020 | 0.233 (0.135, 0.331) | <0.001 | **<0.001** | 0.078 | -0.027 (-0.130, 0.075) | 0.604 | 0.805 | -0.001 |
|  | Model 2 | -0.072 (-0.181, 0.038) | 0.199 | 0.228 | 0.029 | 0.206 (0.102, 0.311) | <0.001 | **<0.001** | 0.104 | -0.052 (-0.167, 0.063) | 0.375 | 0.528 | 0.005 |
| Note: Standardized regression coefficients (β) and FDR-corrected *p* values from linear regression models are presented. Differences significant at FDR-*p* < 0.05 are highlighted in bold. Model 1 was adjusted for sex, age, and standardized total intracranial volume; Model 2 was further adjusted for BMI, hypertension, diabetes, hyperlipidemia, smoking, alcohol consumption, and physical activity (ordinal).  Abbreviations: CI, confidence interval; FDR, false discovery rate. | | | | | | | | | | | | | |
