# Supplementary material for: Mobility-related brain regions linking carotid intima-media thickness to specific gait performances in old age
Source: BMC Geriatr. 2024 Apr 1;24:303. doi: 10.1186/s12877-024-04918-1 (PMC10983675; doi:10.1186/s12877-024-04918-1)
Supplement: Supplementary file 14 — Supplementary Material 14 [file 12877_2024_4918_MOESM14_ESM.docx]

| **Table S13. Mediation effects of mobility-related brain regions in the relation between carotid IMT and specific gait performances.** | | | | | |
| --- | --- | --- | --- | --- | --- |
| Outcome | Mediator | Total effect | ADE | ACDE | Proportion mediated |
| TUG | Primary motor | 0.129 (0.045,0.220)** | 0.076 (-0.018,0.170) | 0.054 (0.020,0.090)*** | 0.415 (0.142,1.310)** |
|  | Sensorimotor | 0.129 (0.045,0.220)** | 0.093 (0.001,0.190) | 0.037 (0.011,0.070)** | 0.284 (0.076,0.980)** |
|  | Visuospatial attention | 0.129 (0.045,0.220)** | 0.112 (0.026,0.200)** | 0.017 (0.003,0.040)* | 0.134 (0.021,0.430)* |
|  | Entorhinal cortex | 0.129 (0.045,0.220)** | 0.082 (-0.018,0.180) | 0.048 (0.016,0.090)** | 0.368 (0.106,1.330)** |
|  | Motor imagery | 0.129 (0.045,0.220)** | 0.095 (0.008,0.190)* | 0.034 (0.012,0.070)*** | 0.266 (0.089,0.850)** |
| Pace | Primary motor | -0.209 (-0.295,-0.140)*** | -0.131 (-0.217,-0.060)*** | -0.078 (-0.124,-0.050)*** | 0.374 (0.220,0.630)*** |
|  | Sensorimotor | -0.209 (-0.295,-0.140)*** | -0.157 (-0.242,-0.080)*** | -0.052 (-0.089,-0.020)*** | 0.250 (0.118,0.460)*** |
|  | Visuospatial attention | -0.209 (-0.295,-0.140)*** | -0.184 (-0.267,-0.110)*** | -0.025 (-0.053,-0.010)** | 0.120 (0.038,0.250)** |
|  | Entorhinal cortex | -0.209 (-0.295,-0.140)*** | -0.164 (-0.258,-0.090)*** | -0.045 (-0.078,-0.020)*** | 0.214 (0.082,0.410)*** |
|  | Motor imagery | -0.209 (-0.295,-0.140)*** | -0.158 (-0.241,-0.090)*** | -0.051 (-0.092,-0.020)*** | 0.243 (0.125,0.420)*** |
|  | Basal ganglia | -0.208 (-0.296,-0.140)*** | -0.183 (-0.270,-0.110)*** | -0.025 (-0.047,-0.010)** | 0.118 (0.034,0.240)** |
| All models were adjusted for sex, age, standardized total intracranial volume, BMI, hypertension, diabetes, hyperlipidemia, smoking, alcohol consumption, and physical activity (polytomous). Values are estimated coefficients (95%CI). The significance threshold was set at **p*<0.05, ***p*<0.01, and ****p*<0.001.  Abbreviations: IMT, Intima-media thickness; TUG, Timed-Up-and-Go; ADE, average direct effect; ACME, average causal mediated effect. | | | | | |
